# Differences and variation in welfare performance of broiler flocks in three production systems

**DOI:** 10.1016/j.psj.2022.101933

**Published:** 2022-04-28

**Authors:** Ingrid C. de Jong, Bram Bos, Jan van Harn, Pim Mostert, Dennis te Beest

**Affiliations:** ⁎ Wageningen Livestock Research, Wageningen University & Research, PO Box 338, 6700 AH, Wageningen, The Netherlands; † Biometris, Wageningen University & Research, P.O. Box 16, 6700 AA, Wageningen, The Netherlands

**Keywords:** animal-based measure, broiler, production system, resource-based measure, welfare assessment

## Abstract

There is a trend toward broiler production systems with higher welfare requirements, that use slower growing broiler strains, apply a reduced stocking density and provide environmental enrichment. Although these separate factors each contribute to increased broiler welfare, there is little information on their combined effect on broiler welfare under commercial conditions, and on the variation in welfare performance of flocks within production systems. The aim of this study was to compare the welfare performance and the between-flock variation in welfare of 3 Dutch commercial broiler production systems differing in welfare requirements: Conventional (C), Dutch Retail Broiler (**DRB**) and Better Life one star (**BLS**). We applied a welfare assessment method based on the Welfare Quality broiler assessment protocol, in which we used 5 animal-based welfare measures collected by slaughterhouses and hatcheries (mortality, footpad dermatitis, hock burn, breast irritation, scratches), and 3 resource- or management-based measures (stocking density, early feeding, environmental enrichment). Data were collected for at least 1889 flocks per production system over a 2-year period. To compare the different measures and to generate an overall flock welfare score, we calculated a score on a scale from 0 to 100 (bad-good) for each measure based on expert opinion. The overall flock score was the sum of the scores of the different welfare measures. The results showed that with increasing welfare requirements, a higher total welfare score was found across production systems (BLS > DRB > C; *P* < 0.0001). Regarding individual measures, C generally had lower (worse) scores than BLS and DRB (*P* < 0.05), except for scratches where C had highest (best) score (*P* < 0.001). Both welfare measure scores and the total welfare score of flocks showed large variation within and overlap between systems, and the latter especially when only the animal-based measures were included in the total flock score. Total flock score ranges including animal-based measures only were: 112.1 to 488.3 for C, 113.0 to 486.9 for DRB, 151.3 to 490.0 for BLS (on a scale from 0 [bad]–500 [good]), with median values of 330.8 for C, 370.9 for DRB, and 396.1 for BLS respectively. This indicates that factors such as farm management and day-old chick quality can have a major effect on the welfare performance of a flock and that there is room for welfare improvement in all production systems.

## INTRODUCTION

There is a societal call towards more welfare-friendly broiler production systems as compared to the conventional production system, especially in North-West Europe (e.g., Bergmann et al., 2016; CIWF, 2018; Saatkamp et al., 2019). The conventional production system is defined as an indoor-only production system using fast-growing broiler strains, usually at stocking densities of 38 kg/m^2^ or higher. In the Netherlands, the market for fresh broiler meat sold by retail changed since 2014 toward broiler meat exclusively produced in broiler production systems using slower-growing broiler chicken strains, housed at reduced stocking densities (<38 kg/m^2^), and provided with environmental enrichment (Saatkamp et al., 2019). Welfare requirements for all fresh meat sold by retail will even be more strict from 2,023 onward. Broiler meat then needs to be produced from production systems using slower growing broiler strains housed at 25 kg/m^2^, and broilers need to be provided with environmental enrichment and a veranda (outdoor area accessible via popholes, covered with a roof and with a mesh wall; Dierenbescherming, 2021). These higher-welfare Dutch production systems claim better broiler welfare than the conventional production system at a moderate increase in production costs (Saatkamp et al., 2019; Vissers et al., 2019), when compared to high-welfare concepts that have already been present for years at a relatively low market share such as organic and free range production. These involve significantly higher production costs than the conventional system (Gocsik et al., 2016). Also other countries, such as Germany (Bergmann et al., 2016) and Denmark (Politiken, 2020), show a trend toward higher-welfare broiler production systems. This trend is likely to continue (CIWF, 2018; Rayner et al., 2020), because some international food businesses have committed themselves to the Better Chicken Commitment. The Better Chicken Commitment requires broiler meat to be produced from systems using slower growing broiler strains at a reduced stocking density as compared to the conventional production system, and providing the chickens with environmental enrichment and natural light.

Studies (partially) based on expert opinion indicate that the change from conventional to higher-welfare systems may improve broiler welfare (Bracke et al., 2019; Stadig, 2019; Vissers et al., 2019). Individual factors applied in these systems, such as slower-growing broiler strains (Dixon, 2020; Rayner et al., 2020), reduced stocking density (Pedersen and Forkman, 2019) and environmental enrichment (reviewed by Riber et al., 2018) may significantly contribute to higher broiler welfare. However, less is known about their combined effect, especially when applied under commercial conditions. For example, there may be an additive effect when these individual factors are combined in higher-welfare systems, and farm management may affect the performance of a flock (de Jong and van Riel, 2020). RSPA (2006), de Jong et al. (summarised in in chapter 6 in Bracke et al., 2020) and Bergmann et al. (2016) showed that broilers in higher-welfare systems had less footpad dermatitis, less hock burn, better gait scores and reduced mortality compared to the conventional system. However, some of these data were collected more than 10 years ago and may be outdated due to developments in genetics and management. In addition, only a limited number of flocks were assessed in these studies, which may neither be representative of the performance of these systems nor give a reliable indication on the variation in welfare between flocks. There is therefore a need for a study comparing the welfare performance and the between-flock variation in welfare in higher-welfare and conventional broiler production systems.

To compare the actual welfare performance of commercial broiler flocks, animal-based measures are preferred (Blokhuis et al., 2013) but these measures, as developed in the Welfare Quality (Welfare Quality, 2009) assessment protocol, and the transect method to assess broiler welfare (BenSassi et al., 2019) require flock visits and are time consuming, costly and not routinely applied in commercial practice. As an alternative Tallentire et al. (2018) used routinely collected data on carcass condemnation, stocking density and mortality to assess flock welfare. This allowed a comparison of large numbers of flocks, but important welfare measures such as contact dermatitis, lameness and behaviors (Bessei, 2006; EFSA, 2012) were lacking in this alternative approach.

The aim of the present study was to explore the welfare performance across, and the between-flock variation in welfare within, different broiler production systems differing in welfare requirements, that is, the conventional broiler production system and 2 higher-welfare production systems. The Welfare Quality protocol (2009) was used as a starting point for a welfare assessment method that included a broad range of welfare measures that were preferably animal-based, and preferably already collected by the sector for quality assurance schemes or legislation. To compare scores between individual measures, and to compare scores between flocks and systems, calculations based on the Welfare Quality method (Welfare Quality, 2009) were done to generate measure scores on a similar scale. Data of at least 1889 flocks per system collected over a 2-year period were used.

## MATERIALS AND METHODS

### Production Systems

Three production systems were included in the dataset and analyzed:

(1) Conventional (C): indoor-only production system (concrete floor with bedding material) using so-called fast-growing broiler chickens with a growth rate above 60 g/d, a slaughter age of maximum 49 d at depopulation of a house, and a maximum stocking density of 42 kg/m^2^ according to the EU Broiler Directive (2007/43/EC), usually without environmental enrichment. Most houses had artificial light only;
(2) Dutch Retail Broiler (DRB): indoor-only production system (concrete floor with bedding material), without natural light entrance, using a slower-growing broiler strain with a maximum growth rate of 50 g/d and housing and management at least according to the ‘Chicken of Tomorrow’ requirements. This involves a maximum stocking density of 38 kg/m^2^ and environmental enrichment (1 bale of straw, hay or other manipulable material/1,000 chickens; Saatkamp et al., 2019; Vissers et al., 2019). There is no requirement regarding slaughter age but in practice this is around 49 days of age;
(3) Better Life System (BLS): production system with an indoor house (concrete floor with bedding material) with at least 3% natural light entrance and in addition a veranda accessible via pop holes (outdoor area with a roof and mesh wall, 20% of the floor area), using a slower-growing broiler strain with a maximum growth rate of 45 g/d and a minimum slaughter age of 56 d, a maximum stocking density of 25 kg/m^2^, and environmental enrichment (2 g of grains per chicken per day and 1 bale of straw, hay or other manipulable material/1,000 broilers), according to the criteria of the Better Life hallmark of the Dutch Animal Protection Organisation (Vissers et al., 2019).

### Welfare Measures

Table 1 summarizes the measures that were included in the welfare assessment. These were based on existing welfare protocols for broilers on-farm, stakeholder and expert input as described in de Jong (2019). Briefly, selection of measures was based on the 4 principles and the underlying criteria as defined in Welfare Quality (Welfare Quality, 2009), but for each principle and criterion literature was screened to determine whether additional or better measures were available, which were preferably animal-based and collected in the production chain because of legislation or quality assurance schemes. In case these were absent, resource-based or management-based measures temporarily served as replacements until feasible animal-based measures are available. For example, as information on behavior was not routinely collected, presence of environmental enrichment, covered veranda or outdoor range and natural light entrance were selected as resource-based indicators, as it is known that these may stimulate species-specific behaviors (Riber et al., 2018; de Jong and Gunnink, 2019; Table 1). Similarly, applications of systems enabling early post-hatch feeding was included as management-based indicator for absence of hunger (Souza da Silva et al., 2021; Table 1) as this information could not be collected yet using an animal-based indicator.

**Table 1:** Measures included in the welfare assessment, whether it is an animal-based or resource- or management-based measure, and the corresponding Welfare Quality principle (Welfare Quality, 2009; de Jong, 2019).

Welfare principle	Animal-based measure	Resource-based or management-based measure
Good feeding		Application of on-farm hatching or hatchery feeding
Good housing		**Maximum stocking density (kg/m**2**)**^1^ (at any time in the production cycle)
Good health	**Proportion of chickens with footpad dermatitis**^1^	
	**Proportion of chickens with hock burn**^1^	
	**Proportion of chickens with breast irritation**^1^	
	Proportion of chickens with scratches and/or wounds	
	**Proportion of mortality (sum of culls and natural death)**^1^	
Appropriate behavior		Provision of environmental enrichment^2^ Presence of covered veranda and/or outdoor range Natural light exposure

1 Bold measures are also included in the Welfare Quality broiler assessment protocol (Welfare Quality, 2009).

2 To be included as environmental enrichment, there should be at least 1 bale/1000 chickens; 2 m of perch/1000 chickens, 0.3 m^2^ of platform area per 100 chickens according to most common commercial guidelines. For other types of enrichment there were no requirements included.

### Data Collection

Data related to the welfare measures as defined in Table 1 were provided by 3 slaughterhouses located in the Netherlands, and collected over a 2-year period (2017 and 2018). Only depopulation data were included in the analysis. Each row in the dataset included data of one flock, a flock being defined as all broilers originating from one broiler house, placed as day-old or as 18-d hatching eggs in case of on-farm hatching. Thus, in case that a farm had more than one broiler house, different flocks could originate from the same farm.

The dataset contained the following information per flock: date of placement of day-old broiler chickens, date of slaughter, anonymous farm code, anonymous flock code, production system, slaughter weight, breed, hock burn (%), breast irritation (%), skin scratches and wounds (%), footpad lesions (% chickens with score 0, 1, and 2), cumulative mortality (%), and whether or not the chickens received early feeding in the hatchery. Production cycle code (each code referring to all flocks slaughtered from one farm that have been placed on the same date as day-old chickens) and age at slaughter were derived from the data and added to the data set. For each welfare measure the method of collection is described in more detail below. In addition, the hatchery provided information whether the chickens hatched traditionally at the hatchery, received early posthatch feeding in the hatchery and have been transported to the broiler farm thereafter, or hatched at the broiler farm. Finally, we added the maximum allowed stocking density (kg/m^2^), the number of the various types of environmental enrichments that were used, and the presence or absence of a covered veranda and natural light exposure in the broiler house to the dataset as described below.

#### Collection of Animal-based Measures

Mortality was registered by the farmers on the food chain information form that accompanied each flock when birds were sent to processing upon depopulation of the house. The mortality figure of a flock comprised all birds found dead and culled, and includes early (first week) mortality and mortality after the first week.

Hock burn data were collected by the slaughterhouse for each depopulated flock, according to the national quality assurance scheme for slaughterhouses (Pluimned, 2021). For each lorry, 100 broilers were scored for the presence of hock burn by inspecting both hocks. Broilers received a score of 1 (presence of hock burn) when a dark colored area of >0.5 cm^2^ on the hock was present and a score 0 if no hock burn was present. As usually multiple lorries are used to transport one flock, multiple samples can be taken per flock.

Breast irritation data were collected by the slaughterhouse for each depopulated flock, according to the national quality assurance scheme for slaughterhouses (Pluimned, 2021). Per lorry, 100 broiler carcasses were scored for the presence of a brown/black area on the breast of 2 cm^2^ or larger, which received a score of 1 (breast irritation present). Broilers received a score 0 when no breast irritation was present.

Data on scratches and wounds were collected by the slaughterhouse for each depopulated flock, according to the national quality assurance scheme for slaughterhouses (Pluimned, 2021). Per lorry, the back and thigh area of 100 carcasses were inspected. A score of 1 (scratches or wound present) was assigned when 3 scratches >2 cm were observed (fresh, or scab, or crust) or when a wound (open skin, either or not covered with a crust) was observed, otherwise broilers received a score 0.

Footpad dermatitis data were collected by the slaughterhouse for each depopulated flock, according to national legislation. Per flock a sample of 100 right feet was collected (50 feet at approximately 1/3 of the flock and 50 feet at approximately 2/3 of the flock) and scored in 3 classes: class 0, no lesion (no or very small discoloration of the feet); class 1, mild lesion (mild lesion, superficial discoloration of the skin, and hyperkeratosis); class 2, severe lesion (epidermis affected, blood scabs, hemorrhage, and severe swelling of the skin; de Jong et al., 2012).

#### Resource- and Management-based Measures

For maximum stocking density, the requirements of the production scheme (for DRB and BLS; Stadig, 2019) and European legislation (Directive 2007/43/EC) and national legislation (for C) were used. Usually, farmers apply the maximum stocking density although it might be slightly lower at slaughter age, dependent on the slaughter date and actual body weight. However, there was no registration of the actual stocking density by the slaughterhouse. This means that all C flocks were assigned a maximum stocking density of 42 kg/m^2^, DRB flocks 38 kg/m^2^, and BLS flocks 25 kg/m^2^.

The presence of environmental enrichment, natural light and a covered veranda was derived from system requirements for DRB and BLS (Stadig, 2019; Vissers et al., 2019). It was noted whether no, 1, 2, or 3 types of enrichments were present; environmental enrichments comprised straw, hay or lucerne bales, pecking stones, scattering grains in the litter, and elevated resting areas (perches, platforms). For C flocks there was no registration whether enrichment or natural light was present (these flocks never have a covered veranda). We thus could not take into account these aspects in our assessment of C farms. It may be that some C flocks were reared with enrichment or natural light, but according to the slaughterhouse enrichment and natural light were not applied by the majority of C farms.

Regarding early feeding, the hatchery recorded per flock whether chickens received feed and water immediately posthatch in the hatchery (and were transported to the farm at day-old), or whether chickens hatched on-farm (18-d incubated eggs were transported to the farm, where the broilers hatched and could access feed and water posthatch) or whether chickens were hatched traditionally (without feed and water until placement of the day-old chickens in the broiler house).

### Calculation of Welfare Measure Scores

According to the existing or new spline functions as described below, welfare measure scores (scale 0–100 from bad to good) were calculated per measure for each flock.

#### Calculating Welfare Scores According to Welfare Quality

For calculation of welfare scores per flock for each measure, the Welfare Quality method (Welfare Quality, 2009) was used as a starting point, which was based on a consultation of experts stating what levels of a welfare measure were considered acceptable and to which degree (using so-called spline functions (Chapter 7 in Blokhuis et al., 2013). Note that we focus here on measure scores rather than the criterion and principle scores in the Welfare Quality model. For each welfare measure, scores were calculated on a scale between 0 and 100, with 0 representing the worst situation and 100 the best according to expert opinion. This enabled us to compare levels of welfare scores for different measures. For 5 of the measures in our database (i.e., footpad dermatitis, hock burn, breast irritation, mortality, and stocking density) a score calculation had been developed by Welfare Quality. We first checked whether the method of data collection has been similar in our study as compared to the Welfare Quality protocol (Welfare Quality, 2009). This was only the case for footpad dermatitis, mortality and stocking density. Note that Welfare Quality classifies footpad dermatitis into 5 classes while we only had 3; class 0 represents no footpad dermatitis; our class 1 was taken to represent mild footpad dermatitis and was considered equivalent to class 1 and 2 in the Welfare Quality protocol. Our class 2 represents severe footpad dermatitis and was considered equivalent to class 3 and 4 in the Welfare Quality protocol. For total mortality including culling, an adjusted calculation was developed by de Jong et al. (2016) and applied in the present study. For stocking density, the Welfare Quality calculation was applied (Welfare Quality, 2009) with the exception that the maximum stocking density included in the formula was set at 42 kg/m^2^ (current EU legislation) instead of 44 kg/m^2^.

#### New Welfare Score Calculations

In case the assessment method of a measure differed as compared to Welfare Quality, or the measure was not included in the Welfare Quality protocol, new score transformation methods were developed. For this, we applied the methodology that was used by Welfare Quality, that is, international experts were consulted and asked to provide graded acceptability scores (using 4 quartile classes ranging from unacceptable, via acceptable and enhanced to excellent as a guide to assign scores on a scale from 0 to 100) for farms with different values for the various measures, and subsequently spline functions were calculated based on the expert opinions (see chapter 7 in Blokhuis et al., 2013 for a description of the methodology).

Eight experts were invited to provide scores. These were from 6 different countries, and were well-known researchers in the field of broiler welfare, housing, and management. Per variable we asked the experts to evaluate a set of 13 to 14 flocks. The evaluated flocks were handpicked based on the distribution of the respective variable in our dataset. Most of the evaluated flocks were located in the range where most of the flocks were located. At the same time we also wanted to cover the observed range without making the distance between 2 consecutive flocks too large. Per variable, we calculated the average across the experts per flock. We then fitted a spline through these averaged values. There are several criteria the spline fit should meet: (1) If the variable has its best score, the spline should return a score of 100, (2) The spline should be monotonously decreasing as the variable of interest is further away from its best value, and (3) the spline function should be strictly positive (e.g., it should not return negative values). We used R (version 3.6.1; R core team, 2019) package *cobs* (version 1.3–3) for fitting of the splines. The advantage of this package is that we can explicitly specify the mentioned 3 fitting criteria. Supplementary File S1 shows the questionnaire as was sent to the experts; Supplementary File S2 shows the graphical representation of the scores as provided by the different experts, and the spline functions that were calculated according to these scores. Scores for early feeding and water, and for enrichment/natural light/covered veranda or outdoor were determined by applying a decision tree as in Welfare Quality (Welfare Quality, 2009). These scores are also included in Supplementary File S2. A software tool in R was developed that can be used to calculate scores for the various measures and a total flock welfare score.

### Calculation of the Total Welfare Score for Broilers On-farm

The overall welfare score per flock (Total Welfare Score, **TWS**) was calculated by summing all scores for the 8 different measures (footpad dermatitis, hock burn, breast irritation, scratches, mortality, stocking density, early posthatch feeding, environmental enrichment, and natural light entrance). The maximum TWS that could be assigned to a flock is then 800 points. An equal weight was assigned to each measure to determine the TWS. According to our opinion there are insufficient arguments in the literature yet to assign a different weighting to each measure to calculate the TWS based on the effect of the individual measure on broiler welfare. Note that assigning a score to an indicator by expert opinion already involves weighting, and that this might change in the future when new research results become available that justifies additional weighting of individual indicators to calculate a TWS.

A further sensitivity analysis of the TWS was performed by omitting the scores for resource-based measures (stocking density, early posthatch feeding, enrichment) one-by-one, in pairs or at all, and comparing these with the total farm score including all measures (always leaving in all 5 animal-based measures, which were: footpad dermatitis, hock burn, breast irritation, scratches, and mortality, **TWS_ABM**).

### Statistical Analysis

Statistical analyses were performed using the software package R (version 3.6.1; R core team, 2019). For each measure we calculated the score per flock as well as the sum of all scores (TWS). Per production system and for both the primary value of the measure (the percentage of chickens with a specific welfare condition), as well as the measure score, TWS and TWS_ABM, the minimum, maximum, 10%, 50%, and 90% percentiles were calculated, in addition to histograms presenting the distributions within and across production systems. Differences in median value between production systems for the primary measure values, measure scores and flock TWS and TWS_ABM were tested with Mood's median test. Mood's median test is nonparametric and tests whether the medians of the 2 systems are identical. In addition, the Wilcoxon rank-sum test was done on the primary measure values and scores. This test compares the sum of ranks of 2 systems and tests whether the 2 systems have a similar distribution.

To further explore the variation in scores within production systems, a boxplot was plotted for each farm which had at least 10 flocks in the dataset. We then calculated the median value per system for the sum of the 5 animal-based measures (TWS_ABM). Farms that had the lower quartile above this median value were marked as being repeatedly better compared to the other farms of the same system. Farms that had the upper quartile below the median value of the type of system as a whole were marked as being repeatedly worse than the other farms.

## RESULTS

### Average Body Weight and Slaughter Age

In the current dataset, C chickens had an average slaughter age of 38.5 ± 4.3 d and weighed 2362 ± 421 gr on average at depopulation. DRB chickens had an average slaughter age of 49.3 ± 1.2 d and weighed 2404 ± 111 gr on average at depopulation, and BLS chickens had an average slaughter age of 56.1 ± 0.6 d and weighed at 2403 ± 107 gr on average at depopulation.

### Primary Values of Welfare Measures and Calculated Welfare Measure Scores per System

Table 2 presents the median and variation for the specific welfare measures and the calculated measure scores and their variation, for the different production systems. Histograms presenting the distribution of the flocks over the measure primary values and welfare measure scores for the 3 production systems are shown in Supplementary File S3. Table 2 shows that, regarding survival (100% minus mortality), C flocks had on average lowest survival (C versus DRB and C versus BLS: *P* < 0.001) and worst calculated score for mortality (C vs. DRB and C vs. BLS: *P* < 0.001), DRB flocks were intermediate and BLS flocks had highest survival and highest scores. A higher prevalence of footpad lesions and thus a lower score was found for C compared to the other systems (C vs. DLB and C vs. BLS: *P* < 0.001). Many C flocks had a score around 20 points for footpad dermatitis whereas most flocks in the other systems were above 80 points, and for C the distribution over the range between 0 and 100 points was more equal than for the other systems (Supplementary File S3). Hock burn scores generally followed the same trend, with higher prevalence and lower score for C than for the other systems (C vs. DRB and C vs. BLS: *P* < 0.001), and BLS having a peak around 100 points (no or very low prevalence of hock burn). Prevalence of breast irritation was low in all systems resulting in more or less similar score distributions although the scores per system differed significantly (C vs. DRB: *P* < 0.05; C vs. BLS: *P* < 0.001; DRB vs. BLS: *P* < 0.001; Table 2). Scratches were more prevalent in BLS and DRB than in C, resulting in a generally higher (better) score for C than for the other production systems (C vs. DRB and C vs. BLS: *P* < 0.001; Table 2). Table 2 and Supplementary File S3 show that there is generally substantial overlap in welfare measure scores between the systems, but the distribution of flocks over the range of scores clearly differs between production systems for all measures except for breast irritation, where nearly all flocks in each production system have low values and thus high scores.

**Table 2:** Prevalence and calculated welfare measure scores for each welfare measure, for C (Conventional; N = 5,683 flocks), DRB (Dutch Retail Broiler; N = 5,936 flocks) and BLS (Better Life system; N = 1,889 flocks) production systems.

Measure	System	Minimum	P0.10	P0.50	P0.90	Maximum
Survival % (100-mortality%)	C	76.6	95.6	97.4^a^	98.4	99.8
	DRB	76.1	95.8	97.8^b^	98.8	100.0
	BLS	87.7	96.9	98.5^c^	99.2	99.9
100- (weighted Footpad lesions %)^1^	C	0	37.4	81.4^a^	98.9	100
	DRB	0	80.3	99.1^b^	100	100
	BLS	4.7	74.3	98.9^b^	100	100
Hock burn %	C	0	0.5	9.0^a^	36.0	88.6
	DRB	0	1.0	5.0^b^	19.5	92.5
	BLS	0	0	2.7^c^	9.0	69.0
Breast irritation %	C	0	0	0^a^	0.2	30
	DRB	0	0	0^b^	0.3	37.7
	BLS	0	0	0^c^	0	24
Scratches %	C	0	0	0.5^a^	2.5	18.0
	DRB	0	0	1.3^b^	3.2	13.5
	BLS	0	0	1.3^c^	4.0	16.5
Stocking density, kg/m^2^^2^	C			42		
	DRB			38		
	BLS			25		
Hatchery fed, % flocks	C			0		
	DRB			1.4		
	BLS			100		
On-farm hatched, % flocks	C			0.7		
	DRB			0.4		
	BLS			0		
One enrichment, % flocks^2^	C			0		
	DRB			100		
	BLS			0		
Two enrichments, % flocks^2^	C			0		
	DRB			0		
	BLS			100		
Three enrichments, % flocks^2^	C			0		
	DRB			0		
	BLS			0		
Veranda, % flocks^2^	C			0		
	DRB			0		
	BLS			100		
Outdoor range, % flocks^2^	C			0		
	DRB			0		
	BLS			0		
Daylight entrance, % flocks^2^	C			0		
	DRB			0		
	BLS			100		

Welfare Measure Score	System	Minimum	P0.10	P0.50	P0.9	Maximum

Mortality score	C	2.2	46.5	66.2^a^	79.3	98.2
	DRB	2.2	46.3	71.4^b^	84.5	99.9
	BLS	3.0	59.6	81.1^c^	89.7	99.2
Footpad lesion score	C	0	13.1	39.4^a^	93.9	100
	DRB	0	37.9	95.4^b^	100	100
	BLS	2.2	31.2	93.9^b^	100	100
Hock burn score	C	2.8	9.9	44.3^a^	98.1	100
	DRB	2.4	20.1	64.6^b^	94.6	100
	BLS	1.1	100	100^c^	100	100
Breast irritation score	C	0.4	78.4	100^a^	100	100
	DRB	0	70.4	100^b^	100	100
	BLS	1.1	100	100^c^	100	100
Scratches score	C	1.4	29.2	84.9^a^	100	100
	DRB	2.7	23.5	57.4^b^	100	100
	BLS	1.7	18.7	56.4^b^	100	100
Stocking density score^2^	C			0		
	DRB			22.3		
	BLS			57.4		
Early feeding score	C	25	25	25	25	81.7
	DRB	25	25	25	25	81.7
	BLS			76.7		
Enrichment score^2^	C			18.2		
	DRB			29.1		
	BLS			77.2		

a,b,c Different superscripts between C, DRB and BLS within a measure or welfare measure score indicate a significant difference between the production systems (*P* < 0.05 at least) tested with Mood's median test. For each measure and calculated welfare measure score the minimum and maximum, and the 0.10, 0.50, and 0.90 percentile are presented. Welfare scores can range from 0 (worst) to 100 (best). Note that values are rounded to one decimal and therefore sometimes appear to be similar, but are not.

1 First, fractions with class 0, 1 and 2 were calculated. Then, the weighted score was calculated according to the formula: 100 - (2 * fraction class 1 * 100 + 7 * fraction class 2 * 100)/7 (see Welfare Quality, 2009) (lower figure meaning more footpad lesions).

2 For these variables, only one value was assigned to the system, which is presented in the column ‘P0.50.’

Thus, significant differences between production systems were found for nearly all measures and scores (see Table 2). Mood's test comparing the median values of the primary welfare measures and welfare measure scores showed that for all measures C significantly differed from DRB and BLS. Median values and scores for the measures for C were lower as compared to the other production systems (*P* < 0.05 at least) except for scratches (*P* < 0.001), which were found more in DRB and BLS. Pairwise comparison of DRB and BLS showed that footpad lesion prevalence and footpad lesion scores did not differ significantly between these systems, whereas the prevalence of scratches differed (*P* < 0.01) but the score did not (i.e., experts did not assign different scores within this range of prevalence). For the other measures DRB significantly differed from BLS (*P* < 0.001), with BLS having better scores than DRB. Wilcoxon signed rank tests generated more or less similar results; only the prevalence of scratches did not differ significantly between DRB and BLS (data not shown). Because the sample sizes were large, these tests have a large power to identify even small differences (see Table 2). The actual difference in median value and the variation for each measure and score is thus likely more informative than the *P*-value of the comparison between production systems.

### Total Welfare Scores and Sensitivity Analysis

**Figure 1:**
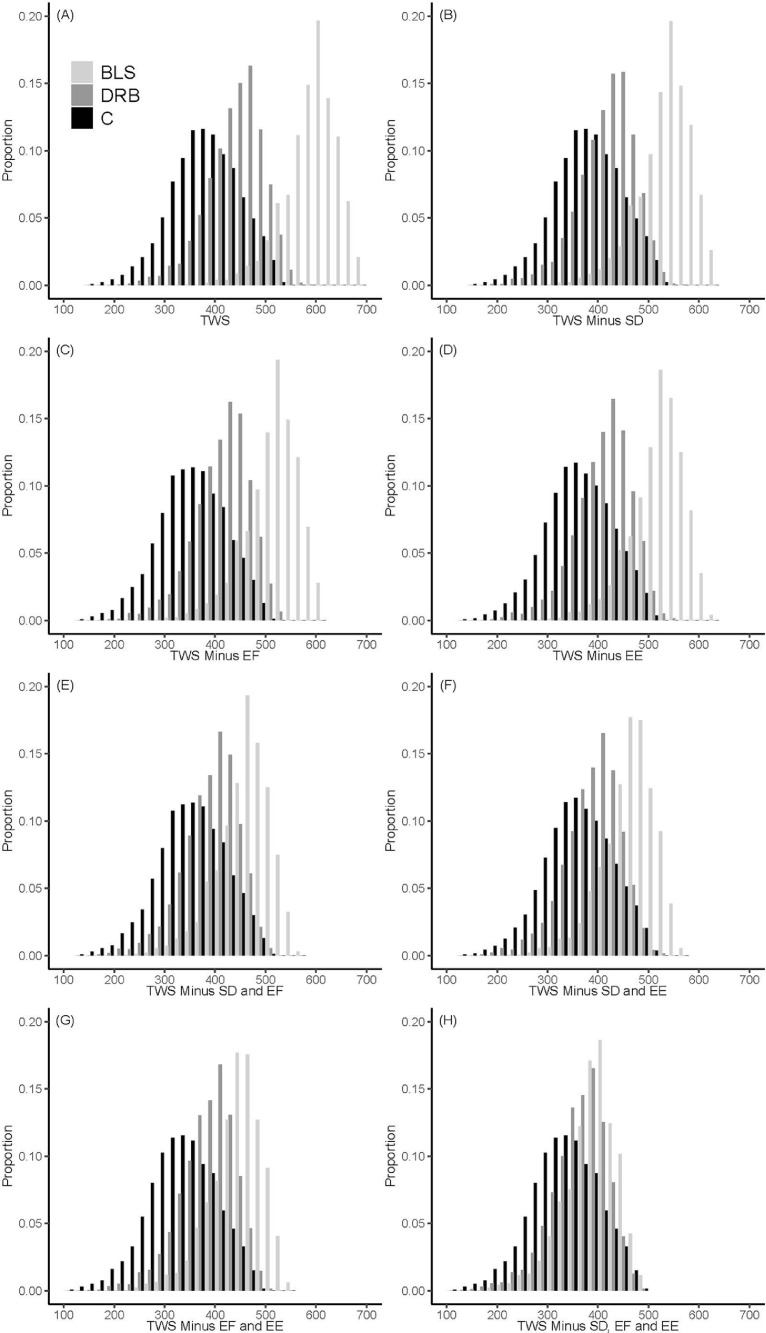
Histogram of the distribution of the Total Welfare Score (TWS) for flocks of the three production systems (histogram A). The TWS for a flock is the sum of the 8 scores for the individual welfare measures. The minimum (worst) score that can be received is 0, maximum and best score is 800. Histograms B-H present the results of the sensitivity analysis and show the TWS minus the scores for Stocking Density (SD) (Histogram B), Early Feeding (EF) (Histogram C), and Environmental Enrichment (EE) (Histogram E) separately. In these graphs the score can range between 0 and 700. Histograms F, G, H show the scores when 2 resource-based measures were omitted (scores ranging from 0 to 600). Histogram H is based on only the 5 animal-based welfare indicators (TWS_ABM) and each flock can therefore only be assigned a score between 0 and 500.

Regarding TWS, a substantial variation in scores between flocks within the 3 production systems was found (Figure 1A). In the current welfare assessment 3 resource-/management-based measures were included (stocking density, early feeding, and environmental enrichment). The respective scores for these measures were identical for all flocks within a particular type of production system for stocking density and enrichment, and variation was low for scores for early feeding (all BLS flocks were hatchery fed while the proportion of on-farm hatching was low for C and DRB) (Table 2). Therefore, a sensitivity analysis was performed by omitting these resource-/management-based measures one-by-one, in pairs and omitting them all; results are shown in Figure 1B to H. The 3 resource-/management-based measures in the current welfare assessment had a relatively high impact on the total welfare score (Figure 1B–H). Omitting these 3 measures resulted in a progressive overlap in scores between the production systems. Table 3 shows the percentiles, minimum and maximum scores per production system for both the total welfare score with 8 variables included, and the scores based on animal based measures only with the 5 animal-based measures included.

**Table 3:** Calculated Total Welfare Score (TWS) for C (conventional), DRB (Dutch Retail Broiler) and BLS (Better Life System).

Production system	Variable^1^	Minimum	P0.10	P0.50	P0.9	Maximum
C	TWS	155.4	288.3	374.4^a^	462.1	531.6
DRB	TWS	189.5	368.9	448.0^b^	505.7	586.3
BLS	TWS	357.6	526.5	602.4^c^	655.8	696.3
C	TWS_ABM	112.1	245.0	330.8^a^	418.3	488.3
DRB	TWS_ABM	113.0	291.9	370.9^b^	427.2	486.9
BLS	TWS_ABM	151.3	320.2	396.1^c^	449.5	490.0

abc Different superscripts within a column indicate significant differences between production systems for TWS, or TWS_ABM respectively (*P* < 0.0001). Scores are presented as the minimum and maximum score, and the 0.10, 0.50 and 0.90 percentile. Total Welfare Score is the score including all 8 measures, Total Welfare Score Animal-Based Measures (TWS_ABM) is the sum of scores excluding stocking density, early feeding and environmental enrichment. Note that the maximum score that can be achieved for a flock is 800 for TWS and 500 for the TWS_ABM.

1 TWS: Total Welfare Score (range 0–800); TWS_ABM: Total Welfare Score for the animal-based measures only (excluding stocking density, early feeding and environmental enrichment) (range 0–500).

In case only the 5 animal-based measures were included (TWS_ABM, Table 3), comparing C with DRB shows that the minimum and maximum values are more or less equal, but that in DRB more flocks have a high score (0.5 percentile is 40 points higher than for conventional), whereas comparing DRB with BLS shows that the minimum level of BLS is higher than DRB and more flocks receive a high score, indicated by the higher median and 0.9 percentile. A significant difference was found between C and DRB, C and BLS, DRB and BLS, both for the TWS as for the TWS_ABM (*P* < 0.0001 for both Mood's and Wilcoxon) (Table 3), thus indicating best welfare for BLS, followed by DRB and C, both when resource- and management-based measures were included and excluded.

### Within Flock Variation per Farm

**Figure 2:**
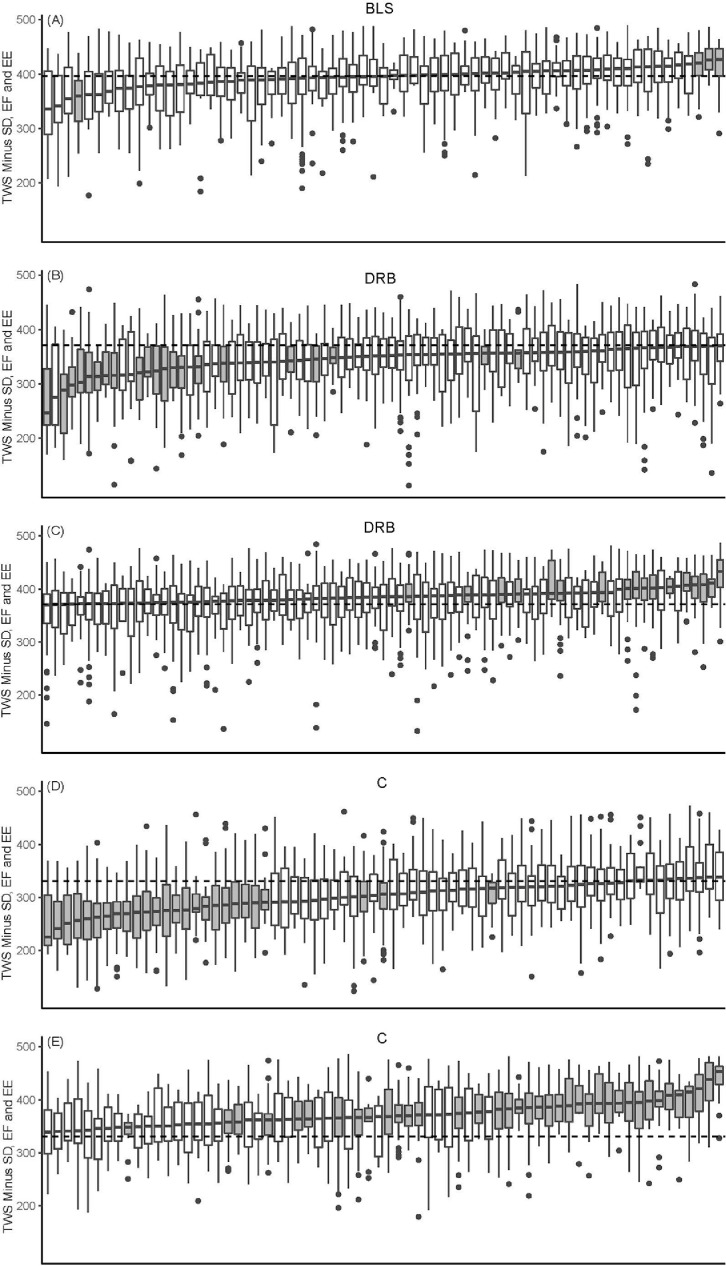
Box plots showing the variation between flocks on a particular farm for the Total Welfare Score based only on animal-based measures (TWS-ABM), that is, without the scores for stocking density, early feeding and environmental enrichment, per production system, for the Better Life System (BLS, upper panel) Dutch Retail Broiler (DRB, second and third panel), and Conventional (C, fourth and fifth panel). Each box plot represents one farm and shows the variation in scores for flocks of one farm, by presenting the median, upper and lower quartile for the Total Welfare Score_ABM and the outliers (dots), where, for this figure, only farms were included with at least 10 flocks over the 2-year study period. Farms that were consistently better or worse as compared to the median score per production system are presented in grey (if these are above the median score these were consistently better, if these are under the median score, these were consistently worse). Median scores were 396.1 for BLS, 370.9 for DRB, and 330.8 for C and are indicated by the dashed horizontal line.

Figure 2 shows the within flock variation per farm per production system for farms with a minimum of 10 flocks within the 2-year period of analysis for the TWS_ABM. The graphs illustrate that both for C and DRB there is considerable variation. Farms can be identified that appeared to be consistently better or worse than the others regarding their welfare performance, that is, the upper or lower quartile is above (better welfare performance) or under (worse welfare performance) the median score of the production system. It also illustrates the lower variation in welfare performance for BLS as compared to DRB and C, and that for BLS only 3 farms could be identified that performed consistently better and one farm that performed consistently worse, where all other farms performed around the median.

## DISCUSSION

The results of the present study showed that the DRB and BLS production systems that have increasing welfare requirements, indeed have a significantly higher TWS as compared to the C production system, and that BLS, with the highest welfare requirements, has the highest score. Scores for single measures showed the same difference, except for scratches where a lower prevalence and better scores were found for the conventional production system (C) as compared to both higher-welfare production systems (BLS and DRB). The results of the present study also showed that there was considerable overlap in welfare scores for the 3 broiler production systems, so, that a flock in a higher-welfare system not necessarily has a better welfare as compared to the conventional system, based on the measures included in our assessment. For 2 production systems (C and DRB), farms could be identified that had consistently worse or better scores compared to the median welfare score of all farms within a production system whereas BLS farms showed a more equal welfare performance.

In the assessment of the different production systems, most measures that have been selected previously (de Jong, 2019) could be included because data were collected by the slaughterhouse and hatchery or (temporarily) replaced by resource-based measures. Only quality of locomotion (gait score), one of the major welfare issues in broiler chickens (Bessei, 2006; EFSA, 2010) and one of the measures of the Welfare Quality broiler assessment protocol (Welfare Quality, 2009), could not be included because of lack of routinely collected data. It is highly recommended to collect gait score data on a routine basis in commercial flocks and to include these data in flock welfare assessments, for example, by application of new technologies (Dawkins et al., 2009; Van Hertem et al., 2018). It may be that inclusion of gait score data would have led to different results, although, based on existing literature, it is expected that systems with slower-grower broiler chickens will have better scores as compared to systems with fast-growing broiler chickens (Bergmann et al., 2016; Dixon, 2020; Rayner et al., 2020), thus BLS and DRB receiving more favorable scores as compared to C.

To our knowledge, this is the first time that data on broiler welfare from a large number of flocks from different production systems were compared. The prevalence of the measures and the average welfare measure scores generated from these data show better welfare and better welfare measure scores for BLS and DRB over C, confirming suggestions based on theoretical analyses (Stadig, 2019), relatively small scale research comparing production systems (RSPCA, 2006; chapter 6 in Bracke et al., 2020; Bergmann et al., 2016; Gerritzen et al., 2019) and expert evaluation of production systems (Bracke et al., 2019). However, analyzing a large number of flocks also illustrates that there is a large variation in welfare measure scores between flocks within the 3 production systems, and that there is considerable overlap in welfare scores across production systems, which is not shown by theoretical studies, expert opinions and small scale studies. Our data show, for example, that TWS_ABM median scores are higher for DRB and BLS as compared to C, but also that the maximum score that is reached is almost similar for the 3 production systems. Moreover, the histograms in Figure 2 illustrate that there is a number of C flocks in the high score range (>400 points), although this number is higher for BLS and DRB. While, on the other hand, there are also DRB and BLS flocks in the low score range (<200 points), although this number is relatively small. Thus, higher welfare requirements are not a guarantee for better flock welfare, and flocks in C systems can also have a good welfare performance.

When comparing the TWS with TWS_ABM, we observed that scores were more overlapping between the 3 production systems when resource-/management-based measures were excluded from the TWS, thus with TWS_ABM. Also the maximum TWS_ABM is close for C, DRB, and BLS, indicating that there are minor welfare differences with optimal management in each system, at least for the included animal-based measures. Indeed, the inclusion of resource-/management-based factors resulted in higher scores of DRS and BLS because these included more strict requirements of stocking density, enrichment and (for BLS) early posthatch feeding as compared to C, which were scored favorably in the expert consultation. Alternatively, it could have been decided to exclude resource-based measures from our welfare assessment, as we did in the sensitivity analysis. However, in that case measures related to broiler behavior were excluded, which we considered an unfavorable situation as it might have led to an unbalanced welfare score. Both negative and positive behaviors are considered important aspects of animal welfare (Dawkins, 2003; Fraser, 2009; Mellor and Beausoleil, 2015). In case these 3 resource-/management-based measures could have been replaced by animal-based measures for both appropriate behavior (use of enrichments, (in)activity, natural behaviors) and absence of hunger, more variation in welfare scores may be found within a production system. This could have resulted in a larger difference in welfare score between C, DRB, and BLS, as it is known that reduced stocking density and environmental enrichment can have positive effects on behavior, for example (Estevez, 2007; Riber et al., 2018), but the actual variation in behavior between flocks is yet not known and remains to be further studied. Likely, with the recent developments in smart farming technologies, these type of data can be collected on a large scale in commercial flocks in the future (Dawkins et al., 2021; Gebhardt-Henrich et al., 2021). This will lead to a more balanced welfare assessment including all dimensions and measured on the animal itself.

The large variation in flock scores within systems suggests that there is room for improvement in all production systems, but more in C and DRB than in BLS, as there was more variation between flock scores in C and DRB than in BLS. It should be noted that in this study more farms with C and DRB were present than with the BLS production system which could have affected this. To further explore the variation within production systems, we compared the TWS_ABM on farm level within each production system. This analysis showed that there are farms performing consistently worse or better as compared to the median score of a system, particularly in C and DRB, whereas in BLS median farm scores were more equal. Although flock performance may vary due to for example, differences in day-old chick quality (Yerpes et al., 2020) and seasonal effects (de Jong et al., 2012; de Jong and van Riel, 2020; Mullan et al., 2021), these data also suggest a large effect of individual farm management on welfare scores, especially in C and DRB. Previous research has shown that differences between individual broiler farms may explain a relatively large proportion of the variation in health and production measures (de Jong and van Riel, 2020). This suggests a management effect, that now also seems to be present in broiler welfare outcomes. Recently, also Mullan et al. (2021) found consistently better or worse farms when comparing welfare and health measures collected from a large number of UK farms. Benchmarking and targeted advise for the worst farms based on practices found in the better farms may lead to a better overall welfare performance within a production system. Interestingly, histograms and box plots indicated that within BLS between-flock and between-farm variation was lower as compared to C and DRB. This might be explained by the more strict welfare requirements in BLS compared to C and DRB, such as the relatively low stocking density and the slower growth rate of the chickens, that may have positive effects on broiler welfare (Martrenchar et al., 1997; Buijs et al., 2009; Knierim, 2013; Dixon, 2020; Rayner et al., 2020) and can make these broilers more resilient towards variation in management practices. Another explanation could be that farmers applying more strict welfare requirements such as BLS are better adopters of good welfare practices.

Animal welfare is increasingly considered to be part of the sustainability of livestock production (Broom, 2019). Here, we assessed broiler welfare by using a much wider range of welfare measures than previously has been used in broiler sustainability assessments methods (Castellini et al., 2012; Tallentire et al., 2018), and that is based on actual, routinely collected data in the production chain instead of on literature (Bokkers and de Boer, 2009; van Wagenberg et al., 2017). In these other studies, various methods have been used to assign a single score to animal welfare within a sustainability assessment (Castellini et al., 2012; van Asselt et al., 2015; Tallentire et al., 2018) and there is not a single generally applied method yet. In these papers, stakeholders or experts were consulted to assign scores to measures. By applying the Welfare Quality method, we used expert opinion to assign scores to prevalence of measures which, similar as in previous studies, involves subjectivity. This subjectivity was considered acceptable as long as the method is transparent and sufficiently sensitive to variation in individual welfare measure scores (Sandoe et al., 2019), and it is a way to generate a total welfare score for a flock which is based on several measures with a different scaling. However, it should be taken into account that the selection of experts determines the final outcome. Including other stakeholders may lead to different weighting and thus a different scoring system (Sandoe et al., 2019). A critical review of the Welfare Quality method to calculate flock scores led to the suggestion that a scoring system should at least (1) make sure that serious welfare problems are not overlooked or underestimated, and (2) be transparent with respect to the ethical decisions made (Sandoe et al., 2019). We tried to meet these points in the present score calculations, but our system can be improved by at least including an indicator for quality of locomotion. Our approach is considered as a possible method to include animal welfare in the overall sustainability assessment of broiler production systems. It includes the most important welfare issues in broiler chickens (Bessei, 2006; EFSA, 2010; 2012), and it provides insight in differences between flocks with respect to the selected measures. Furthermore, such a welfare assessment method should not be considered as static but should be updated as soon as new developments in the field are available, for example, methods to assess lameness on a routine basis. Any update may also include automation of measurement so that possible subjectivity in data collection could be excluded.

In Welfare Quality, after generating scores for the individual measures, an additional expert weighting is done to calculate criterion, principle and the total welfare score so that some measures received higher weights, thus had a larger effect, on the total score. However, this resulted in a scoring system that appeared to be insensitive to the actual difference on the level of welfare measures, where only 2 measures (stocking density and drinker space) determined 95% of the overall classification of a flock (Buijs et al., 2017). To prevent this, we decided to only include an expert weighting to generate scores from the various measures on the same scale (0–100), and to give all measures a similar weight to generate the TWS (i.e., simple summing the individual scores). Inspection of the histograms with the flock scores per system showed a considerable variation between flocks within a system, and between the production systems, indicating that a simple summing of scores could be sufficiently sensitive to provide insight in the actual differences between flocks. However, assigning a different weight to measures, for example, to measures that are perceived to have more serious effects on welfare, could have generated a different outcome and provides an opportunity to assign more weight to measures that are more important with respect to broiler welfare. We have considered this option, but based on the existing literature on the welfare effects of the single measures we considered it very difficult to assign different weights to the measures. We therefore did not include this in the present score calculation. Another drawback of the choice to simply sum up the measure scores to one TWS is that compensation is allowed; that is, a very low score for a certain measure can be compensated by a number of higher scores of other measures. This was also one of the criticisms regarding Welfare Quality (Sandoe et al., 2019). This can be overcome by for example, reducing the total flock score when a certain threshold for a measure is reached, as was done in Jacobs et al. (2017) in an assessment method to determine welfare in the end-of life stage of broiler chickens, or by indicating minimum values that need to be obtained for each of the measures. This can also be part of a follow up study to further refine the present welfare score calculation.

In conclusion, our results showed that production systems with higher welfare requirements (BLS and DRB) indeed led to an on average improved level of welfare, also when resource-based measurements were excluded from the calculation of the total welfare score. For most individual animal-based measures DRB and BLS also performed better than C, but not for scratches, which were less observed in C flocks. However, our results also showed that within each system there was a high variation in flock welfare scores, and that this variation was larger within C and DRB than within BLS. These results also indicate that within each of the three production systems there is room for improvement of welfare.

## Appendix Supplementary materials

Image, application 1

Image, application 2

Image, application 3
