# Supplementary material for: Differences and variation in welfare performance of broiler flocks in three production systems
Source: Poult Sci. 2022 Apr 28;101(7):101933. doi: 10.1016/j.psj.2022.101933 (PMC9189189; doi:10.1016/j.psj.2022.101933)
Supplement: Supplementary file 1 [file mmc1.docx]

**Supplementary file S1**

Questionnaire sent to experts

**Note: Bruises and the number of animals dead-on-arrival were excluded from the present paper and therefore also from the questionnaire below.**

**Introduction**

Wageningen Livestock Research, together with four partners in the broiler production chain and the Ministry of Agriculture, Nature and Food Quality, is currently developing a sustainability assessment model for broiler chicken production within the ‘Greenwell’ project. This sustainability assessment model consists of three sub-models, i.e. a welfare assessment model, an environmental impact model, and an economical model.

Regarding the welfare assessment model, the Greenwell project chose to base the model on the existing Welfare Quality® assessment protocol for broiler chickens (on-farm stage)^^[[1]](#footnote-1)^^. Regarding the end-of-life stage, the hatchery stage and the broiler breeder stage, new models have been developed, based on the welfare criteria and measures as defined by Welfare Quality®, but with specific measures regarding the stage of the production chain. If present, existing models, such as WellTrans^^[[2]](#footnote-2)^^ for the end-of-life stage, were taken into account^1^.

A few new animal-based indicators were considered by the Greenwell consortium for the assessment of broiler welfare on-farm and are included in final selection of key-indicators. Reasons for adding new indicators in addition to indicators of existing models is that in the existing Welfare Quality^®^ protocol, indicators for some criteria are lacking (e.g., for social behaviour), or are subject to discussion between scientists because of lack of validity (e.g., Qualitative Behaviour Assessment and Touch Test in the Welfare Quality^®^ broiler assessment protocol^^[[3]](#footnote-3)^^), and that new indicators of animal welfare are still being developed.

Further, as Greenwell will use data collected in the broiler chain, there are sometimes differences in the scoring method as compared to Welfare Quality®, which requires that the current function to calculate an indicator score, as described by Welfare Quality, should be adjusted. An example is the scoring for hock burn, which has five classes according to Welfare Quality®, but only two classes if done by the plant, due to the high slaughter line speed.

*Indicators requiring new calculations for indicator scores*

The following indicators for broiler welfare on-farm and during the end-of-life stage need adjustment of the spline function to calculate the indicator score, or a spline function needs to be developed because the indicator is new (Table 1):

**Table 1** : list of indicators for which a new calculation is required in Greenwell, with reason.

| **Indicator** | **Reason** |
| --- | --- |
| Early feeding (post-hatch) | New indicator, relates to absence of hunger and thirst |
| Hock burn | Scoring method at the plant differs from Welfare Quality® |
| Breast burn/irritation | Replaces breast blisters, as these are hardly observed in broiler chickens; spline calculation in the Welfare Quality® protocol needs to be adjusted. |
| Scratches | New indicator, relates to absence of injuries |
| Presence of enrichment/outdoor range/natural light | New indicator; this resource-based measure will temporarily replace the animal-based measure ‘species specific behaviour’ until these data can/will be collected on-farm |
| Bruises | Indicator for welfare during end-of-life stage, no calculation developed by Welfare Quality® |
| Dead-on-arrival | Indicator for welfare during end-of-life stage, no calculation developed by Welfare Quality® |

*Methodology*

Because we will use the Welfare Quality® calculation of scores for the indicators, we would like to follow the same methodology for the (additional) indicators in the Greenwell welfare model for broilers on-farm. This enables us to calculate a final welfare score for broiler flocks of different production systems. Please note that we will only calculate scores per indicator, and that at this moment we will not generate criterion and principle scores as is done in the Welfare Quality® assessment protocol.

*Expert consultation*

We would like to ask your help to define functions to calculate scores for indicators that have been added in the Greenwell welfare assessment model. An appropriate method would be to generate spline functions for the indicators % chickens with scratches, hock burn, breast burn/irritation, bruises, and % dead-on-arrival. A decision tree will be applied to calculate a score for the indicators ‘early feeding’ and ‘enrichment/natural light/veranda/outdoor range’, according to the approach of Welfare Quality®. In order to do this, we need an additional expert opinion on each of these indicators.

*Instructions to fill in the tables*

In the current document, you will find tables with virtual data with regard to the total prevalence of birds with injuries, hock burn, breast burn/irritation, bruises and the prevalence of dead-on-arrival. Further, for your information, a histogram is presented showing the distribution of the indicator prevalence for a one-year period as collected at slaughter. This includes different farm types, i.e., conventional farms with fast growing strains and farms with slower growing broilers, lower stocking densities and environmental enrichment. You are kindly asked to keep this information confidential. Further, you will find two decision trees regarding the presence or absence of early feeding and environmental enrichment/outdoor range.

For each indicator I would like to ask you to give a score for the level of welfare for each of the virtual farms (see tables below). The welfare score is always between 0 and 100; a score of 0 refers to the lowest level of welfare, and a score of 100 refers to the highest possible level of welfare. As a point of reference, you are advised to keep in mind that a welfare score below 20 refers to an unacceptable situation, a score between 20 and 50 would be just acceptable, a score between 50 and 80 would refer to an enhanced welfare situation, and in case of a score > 80, the welfare situation would be considered excellent.

Similarly, I would like to ask you to assign a score for either or not using early feeding, and the different combinations of natural light/enrichment/outdoor range.

If you feel that you do not have the expertise to fill in one of the tables, please indicate this in your email and leave it empty.

Your names will not be disclosed, we will just mention the number of experts that contributed to this consultation.

Thank you very much for your collaboration. If necessary, you can always contact me at [contact information]

1. **Hock burn**

Please assign a score to the prevalence of hock burn on the 14 virtual farms as shown in Table 2. Please keep in mind the meaning of the scores as indicated below the table. As a reference, a histogram of the prevalence of hock burn during a one year period for different types of broiler farms (conventional to higher-welfare systems) is presented below the table.

Hock burn is scored as follows^^[[4]](#footnote-4)^^:

Score 0: no evidence of hock burn, or a discoloration of a size of 0.5 cm^2^  or less

Score 1: evidence of hock burn, any brown or black discoloration of the hock of at least 0.5 cm^2^

**Table 2:** virtual dataset and scoring table for hock burn.

| **Farm** | **Prevalence of hock burn (%)** | **Score (100=perfect)** |
| --- | --- | --- |
| 1 | 0 |  |
| 2 | 1 |  |
| 3 | 2 |  |
| 4 | 3 |  |
| 5 | 4 |  |
| 6 | 6 |  |
| 7 | 8 |  |
| 8 | 11 |  |
| 9 | 15 |  |
| 10 | 21 |  |
| 11 | 30 |  |
| 12 | 50 | 0 |
| 13 | 70 | 0 |
| 14 | 100 | 0 |

| **Meaning of scores:** | | | |
| --- | --- | --- | --- |
| 0 unacceptable | 20 acceptable | 55 enhanced | 80 excellent 100 |

**Figure 1:** histogram of prevalence of hock burn (score 1) during 1 year, in different types of broiler systems.

1. **Breast burn/irritation**

Please assign a score to the prevalence of breast burn/irritation on the 14 virtual farms as shown in Table 3. Please keep in mind the meaning of the scores is indicated below the table. As a reference, a histogram of the prevalence of breast burn during a one year period for different types of broiler farms (conventional to higher-welfare systems) is presented below the table.

Breast burn is scored as follows^[[5]](#footnote-5)^:

Score 0: no evidence of breast burn/irritation or discoloured area/lesion smaller than 0.5 cm^2^

Score 1: A brown/black discoloured area or lesion larger than 0.5 cm^2^ on the breast

**Table 3:** virtual dataset and scoring table for breast burn/irritation.

| **Farm** | **Prevalence of breast burn/irritation (%)** | **Score (100=perfect)** |
| --- | --- | --- |
| 1 | 0 |  |
| 2 | 0.1 |  |
| 3 | 0.2 |  |
| 4 | 0.3 |  |
| 5 | 0.4 |  |
| 6 | 0.6 |  |
| 7 | 0.9 |  |
| 8 | 1.5 |  |
| 9 | 3.0 |  |
| 10 | 5.0 |  |
| 11 | 9.0 |  |
| 12 | 15.0 |  |
| 13 | 25.0 | 0 |
| 14 | 40.0 | 0 |

| **Meaning of scores:** | | | |
| --- | --- | --- | --- |
| 0 unacceptable | 20 acceptable | 55 enhanced | 80 excellent 100 |

**Figure 2:** histogram of prevalence of breast burn (score 1) during 1 year, in different types of broiler systems.

1. **Scratches**

Please assign a score to the prevalence of scratches on the 13 virtual farms as shown in Table 4. Please keep in mind the meaning of the scores as indicated below the table. As a reference, a histogram of the prevalence of scratches during a one year period for different types of broiler farms (conventional to higher-welfare systems) is presented below the table.

Scratches are scored as follows^[[6]](#footnote-6)^:

Score 0: no evidence of scratches, or less or smaller than defined for score 1 on the breast or thigh area

Score 1: a score of 1 was assigned when 3 scratches > 2cm were observed (fresh, or scab or crust) or when a wound (open skin, either or not covered with a crust) was observed on the breast or thigh area

**Table 4**: virtual dataset and scoring table for scratches.

| **Farm** | **Prevalence of scratches** | **Score (100=perfect)** |
| --- | --- | --- |
| 1 | 0 |  |
| 2 | 0.4 |  |
| 3 | 0.8 |  |
| 4 | 1.2 |  |
| 5 | 1.6 |  |
| 6 | 2.0 |  |
| 7 | 2.8 |  |
| 8 | 4.0 |  |
| 9 | 6.0 |  |
| 10 | 8.0 |  |
| 11 | 11.0 |  |
| 12 | 15.0 |  |
| 13 | 20.0 |  |

| **Meaning of scores:** | | | |
| --- | --- | --- | --- |
| 0 unacceptable | 20 acceptable | 55 enhanced | 80 excellent 100 |

**Figure 3:** histogram of prevalence of scratches (score 1) during 1 year, in different types of broiler systems.

1. **Early feeding**

It has been shown that feed deprivation of 36 hours or more after hatching increases the risk for mortality in later life. Although the yolk sac provides energy during the first hours post-hatch, newly hatched chicks may suffer from hunger and thirst as it may take long before they receive their first feed and water, depending on e.g. hatching moment (early or late hatchers) and transport time^^[[7]](#footnote-7)^^. To prevent hunger and thirst due to feed and water deprivation post-hatch, broilers can be fed in the hatchery and transported to the farm afterwards, or can hatch in the broiler house (in that case 18-days incubated eggs are placed in the broiler house). Currently, less than 10% of the broilers hatch in the house in the Netherlands and a small proportion of chickens receives feed in the hatchery.

Please assign a score to the different early feeding systems post-hatch as indicated in the decision tree.

**Decision tree 1.** Different systems for early feeding (click on the cell to fill in the score).

| **Meaning of scores:** | | | |
| --- | --- | --- | --- |
| 0 unacceptable | 20 acceptable | 55 enhanced | 80 excellent 100 |

1. Natural light and environmental enrichment

In the ideal situation, the behaviour of broiler chickens is scored and included in the welfare assessment. However, there are practical limitations to collect these data in practice, although in the future this might be possible due to development of e.g. sensor techniques. Therefore, we included the presence or absence of resources such as natural light, environmental enrichment and (outdoor) ranges in the assessment model, until we would be able to record broiler behaviour in commercial flocks.

Currently in commercial systems in the Netherlands, there is variation in the application of natural light, the presence of a range or veranda, and the number of environmental enrichment items that is provided. The latter can be of different types, such as perches, platforms, pecking objects (pecking stones) and bales. Sometimes several enrichment types are present, e.g. elevated resting place and pecking objects. We ask you to assign a score to the different combinations of natural light, range and environmental enrichment as indicated below. For clarity, the decision tree is presented as a table.

| **Natural light inside the house** | **Veranda/outdoor** | | **Number of additional enrichment types in the house** | | **Score (100=perfect)** | |
| --- | --- | --- | --- | --- | --- | --- |
| No | No | | 0 | |  | |
|  |  |  | 1 | |  | |
|  |  |  | 2 | |  | |
|  |  |  | ≥ 3 | |  | |
|  | Covered veranda | | 0 | |  | |
|  |  |  | 1 | |  | |
|  |  |  | 2 | |  | |
|  |  |  | ≥ 3 | |  | |
|  | Outdoor range | | 0 | |  | |
|  |  |  | 1 | |  | |
|  |  |  | 2 | |  | |
|  |  |  | ≥ 3 | |  | |
|  | Covered veranda AND outdoor range | | 0 | |  | |
|  |  |  | 1 | |  | |
|  |  |  | 2 | |  | |
|  |  |  | ≥ 3 | |  | |
| Yes | No | | 0 | |  | |
|  |  |  | 1 | |  | |
|  |  |  | 2 | |  | |
|  |  |  | ≥ 3 | |  | |
|  | Covered veranda | | 0 | |  | |
|  |  |  | 1 | |  | |
|  |  |  | 2 | |  | |
|  |  |  | ≥ 3 | |  | |
|  | Outdoor range | | 0 | |  | |
|  |  |  | 1 | |  | |
|  |  |  | 2 | |  | |
|  |  |  | ≥ 3 | |  | |
|  | Covered veranda AND outdoor range | | 0 | |  | |
|  |  |  | 1 | |  | |
|  |  |  | 2 | |  | |
|  |  |  | ≥ 3 | |  | |
| **Meaning of scores:** | | | | | |  |
| 0 unacceptable | | 20 acceptable | | 55 enhanced | 80 excellent 100 |  |

1. De Jong, I.C., 2019. Development of the ‘animal welfare’ dimension within the Greenwell sustainability assessment model: 1. justification of the selection of indicators. Wageningen Livestock Research Report 1194. [↑](#footnote-ref-1)
2. JACOBS, L., DELEZIE , E., GOETHALS, K., AMPE, B., DUCHATEAU, L. & TUYTTENS, F. A. M. 2017. Vleeskippenwelzijn tijdens de pre-slachtfase Evaluatieprotocol en Online integratie-tool. Melle, Belgium: ILVO. [↑](#footnote-ref-2)
3. The relationship between fear of human and lameness in broilers (2018). Vasdal, G., de Jong, I., Moe, R. O., Granquist, E.G. Animal, 12, 334-33910.1017/s1751731119000466 [↑](#footnote-ref-3)
4. https://pluimned.avined.nl/sites/pluimned/files/na_1_mrt_17_8-beoordelingssysteem_vleeskuikens_ikb_kip_-_versie_4_-_170301.pdf [↑](#footnote-ref-4)
5. https://pluimned.avined.nl/sites/pluimned/files/na_1_mrt_17_8-beoordelingssysteem_vleeskuikens_ikb_kip_-_versie_4_-_170301.pdf [↑](#footnote-ref-5)
6. https://pluimned.avined.nl/sites/pluimned/files/na_1_mrt_17_8-beoordelingssysteem_vleeskuikens_ikb_kip_-_versie_4_-_170301.pdf [↑](#footnote-ref-6)
7. DE JONG, I. C., VAN RIEL, J., BRACKE, M. B. M. & VAN DEN BRAND, H. 2017. A 'meta-analysis' of effects of post-hatch food and water deprivation on development, performance and welfare of chickens. *PLoS One,* 12**,** e0189350. [↑](#footnote-ref-7)
