# Supplementary material for: Differences and variation in welfare performance of broiler flocks in three production systems
Source: Poult Sci. 2022 Apr 28;101(7):101933. doi: 10.1016/j.psj.2022.101933 (PMC9189189; doi:10.1016/j.psj.2022.101933)
Supplement: Supplementary file 2 [file mmc2.docx]

**Supplementary File S2.**

Graphical representations of expert scores and spline functions for welfare measures where new functions were developed for, and decision tree scores.

**
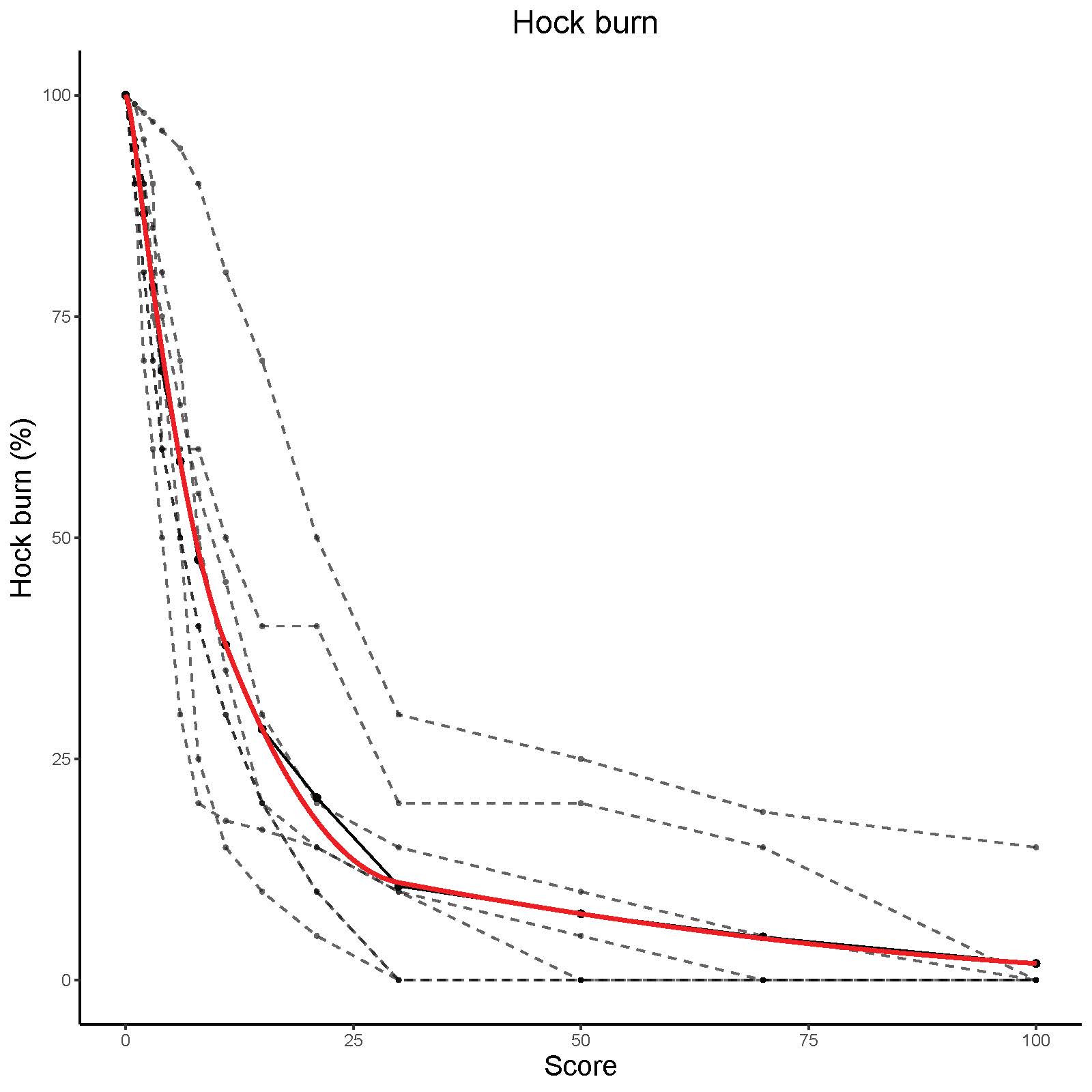
**

**Figure 1.** Expert scores (dashed lines) and calculated spline function (red solid line) for hock burn.

**
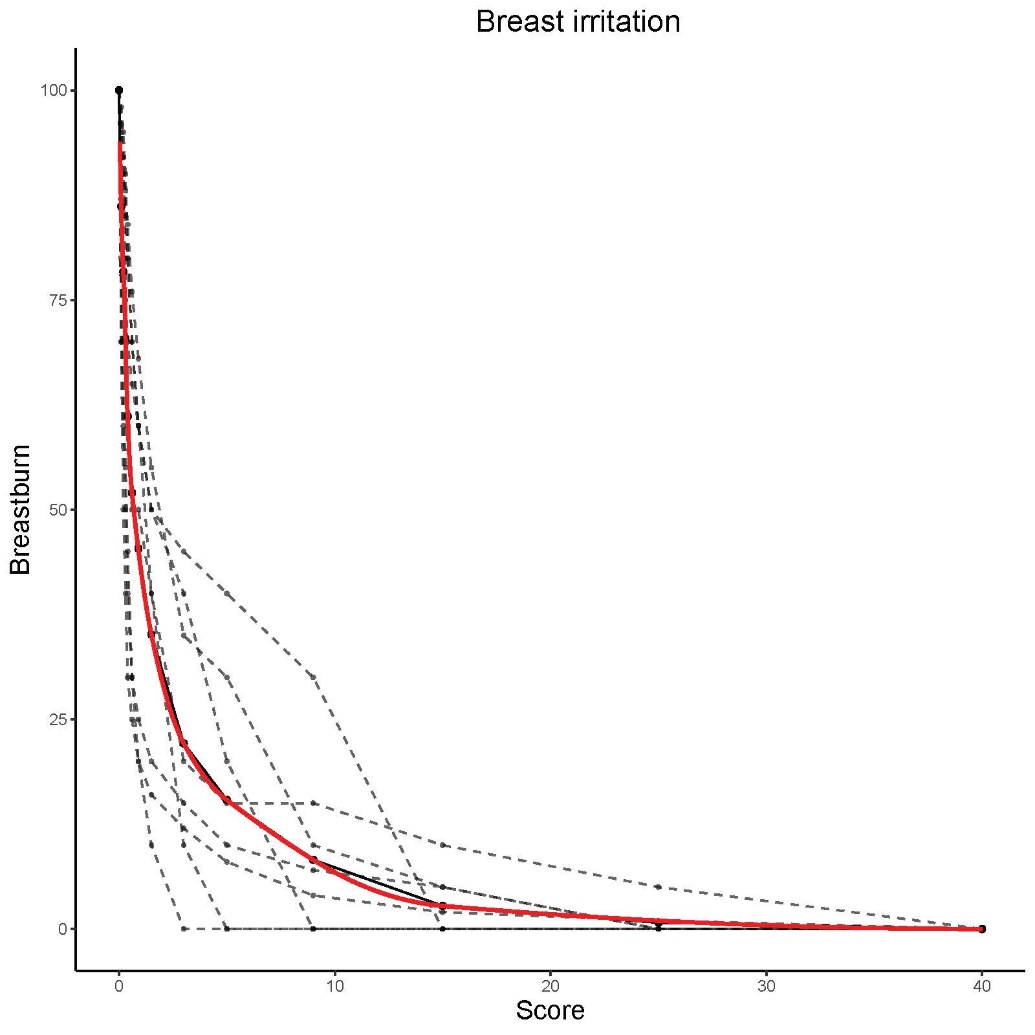
**

**Figure 2.** Expert scores (dashed lines) and calculated spline function (red solid line) for breast burn.

**
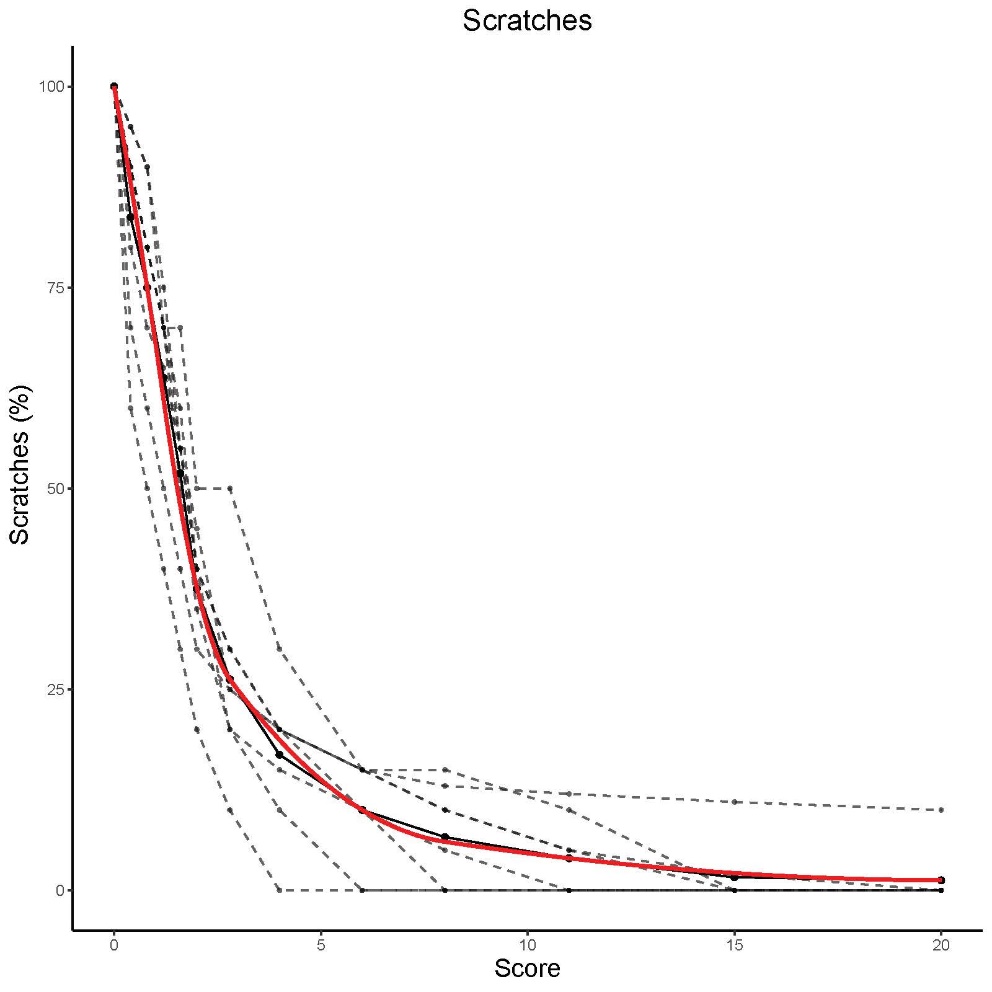
**

**Figure 3.** Expert scores (dashed lines) and calculated spline function (red solid line) for scratches and wounds.

**Table 1.** Decision tree showing the individual expert scores (experts 1-9) and the average score for the absence or presence of early feeding. Note that that two experts did not assign scores to this indicator.

| Expert | 1 | 2 | 3 | 4 | 5 | 6 | score |
| --- | --- | --- | --- | --- | --- | --- | --- |
| Early feeding hatchery | 70 | 55 | 100 | 65 | 100 | 70 | **76.7** |
| On-farm hatching | 100 | 80 | 50 | 100 | 60 | 100 | **81.7** |
| No early feeding | 30 | 20 | 30 | 20 | 0 | 50 | **25.0** |

**Table 2.** Decision tree showing the individual expert scores and the average score for environmental enrichment, natural light and veranda/outdoor range.

| Natural light inside the house | Veranda/  outdoor | Number of additional enrichment types in the house | 1 | 2 | 3 | 4 | 5 | 6 | 7 | 8 | Score |
| --- | --- | --- | --- | --- | --- | --- | --- | --- | --- | --- | --- |
| No | **No** | **0** | 20 | 0 | 20 | 40 | 21 | 0 | 0 | 45 | **18.2** |
|  |  | **1** | 25 | 20 | 30 | 55 | 23 | 20 | 10 | 50 | **29.1** |
|  |  | **2** | 30 | 40 | 35 | 70 | 25 | 55 | 20 | 55 | **41.2** |
|  |  | **≥ 3** | 35 | 50 | 40 | 80 | 27 | 80 | 40 | 60 | **51.5** |
|  | **Covered veranda** | **0** | 35 | 55 | 30 | 70 | 64 | 20 | 30 | 60 | **45.5** |
|  |  | **1** | 40 | 60 | 40 | 70 | 66 | 55 | 40 | 65 | **54.5** |
|  |  | **2** | 45 | 75 | 40 | 80 | 68 | 75 | 50 | 70 | **63.9** |
|  |  | **≥ 3** | 50 | 75 | 50 | 90 | 70 | 90 | 60 | 75 | **70.0** |
|  | **Outdoor range** | **0** | 65 | 55 | 40 | 70 | 74 | 20 | 50 | 70 | **55.5** |
|  |  | **1** | 70 | 65 | 50 | 80 | 76 | 55 | 60 | 75 | **66.4** |
|  |  | **2** | 75 | 75 | 55 | 90 | 78 | 75 | 70 | 80 | **74.7** |
|  |  | **≥ 3** | 80 | 75 | 60 | 90 | 80 | 90 | 80 | 85 | **80.0** |
|  | **Covered veranda AND outdoor range** | **0** | 80 | 60 | 40 | 60 | 84 | 20 | 60 | 80 | **60.5** |
|  |  | **1** | 85 | 75 | 60 | 70 | 86 | 55 | 70 | 85 | **73.2** |
|  |  | **2** | 90 | 80 | 70 | 90 | 88 | 75 | 80 | 90 | **82.9** |
|  |  | **≥ 3** | 95 | 80 | 80 | 90 | 90 | 90 | 90 | 95 | **88.7** |
| Yes | **No** | **0** | 25 | 15 | 25 | 70 | 51 | 0 | 10 | 50 | **30.7** |
|  |  | **1** | 30 | 40 | 30 | 80 | 53 | 20 | 20 | 55 | **41.0** |
|  |  | **2** | 35 | 55 | 30 | 80 | 55 | 60 | 30 | 60 | **50.6** |
|  |  | **≥ 3** | 40 | 55 | 50 | 90 | 57 | 80 | 50 | 65 | **60.9** |
|  | **Covered veranda** | **0** | 40 | 50 | 40 | 80 | 74 | 25 | 40 | 65 | **51.7** |
|  |  | **1** | 55 | 60 | 45 | 80 | 76 | 60 | 50 | 70 | **62.0** |
|  |  | **2** | 60 | 80 | 55 | 90 | 78 | 80 | 60 | 75 | **72.2** |
|  |  | **≥ 3** | 65 | 80 | 60 | 100 | 80 | 100 | 70 | 80 | **79.4** |
|  | **Outdoor range** | **0** | 70 | 60 | 45 | 80 | 84 | 25 | 60 | 75 | **62.4** |
|  |  | **1** | 75 | 80 | 70 | 90 | 86 | 60 | 70 | 80 | **76.4** |
|  |  | **2** | 80 | 85 | 90 | 90 | 88 | 80 | 80 | 85 | **84.7** |
|  |  | **≥ 3** | 85 | 85 | 100 | 90 | 90 | 100 | 90 | 90 | **91.2** |
|  | **Covered veranda AND outdoor range** | **0** | 85 | 75 | 45 | 70 | 94 | 25 | 70 | 85 | **68.6** |
|  |  | **1** | 90 | 100 | 80 | 90 | 96 | 60 | 80 | 90 | **85.7** |
|  |  | **2** | 95 | 100 | 100 | 100 | 98 | 80 | 90 | 95 | **94.7** |
|  |  | **≥ 3** | 100 | 100 | 100 | 100 | 100 | 100 | 100 | 100 | **100.0** |
