# Supplementary material for: Differences and variation in welfare performance of broiler flocks in three production systems
Source: Poult Sci. 2022 Apr 28;101(7):101933. doi: 10.1016/j.psj.2022.101933 (PMC9189189; doi:10.1016/j.psj.2022.101933)
Supplement: Supplementary file 3 [file mmc3.docx]

**Supplementary File S3**


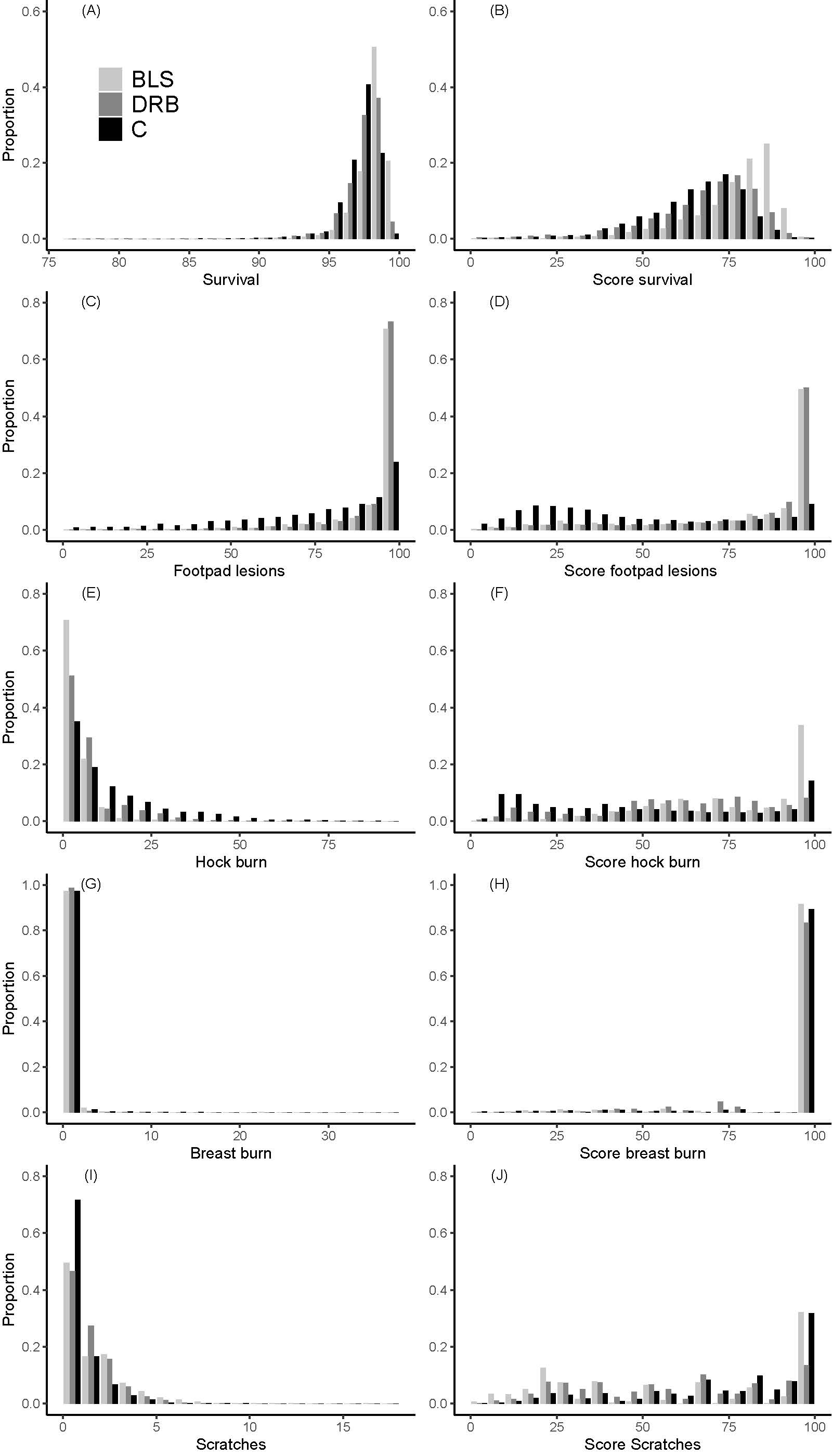


Figure. Histograms of the prevalence and welfare measure scores for the animal-based measures for the three production systems (A, B: survival; C,D: footpad lesions; E, F: hock burn; G, H: Breast burn; I, J: scratches). No histograms are presented for early feeding, stocking density and enrichment/natural light/veranda or outdoor range as these have only one value per production system or are expressed as percentage per production system (early feeding).
